# Validation of Advanced Paediatric Life Support Formulas for Weight Calculation in a Multiethnic Population

**DOI:** 10.5402/2012/869634

**Published:** 2012-09-25

**Authors:** Colette Seddon, Laurell Lockitt, Sacha Dhanjal, Michael Eisenhut

**Affiliations:** Luton & Dunstable Hospital NHS Foundation Trust, Lewsey Road, Luton LU40DZ, UK

## Abstract

*Introduction*. The aims of this study were to validate the new formulas for weight calculation introduced by the advanced life support group (alsg) of the United Kingdom in 2011 and compare their performance to the formula currently used by the European Resuscitation Council (ERC) and other formulas and to check whether performance of formulas for weight calculation is affected by ethnic group and gender. *Methods*. Prospective audit of weight versus calculated weight comparing alsg formula with ERC, Luscombe, Argall, and Best Guess formulas analysed for gender, age, and ethnic groups. *Results*. Prospectively 599 children were included: 157 Asian, 268 Caucasian, and 174 children from other origin. In infants there was no difference between actual weight and alsg formula calculated weight. There was a progressively increased underestimation of weight year by year from 1 to 10 years of age using the ERC formula. In the 6–10 year age group the ERC formula underestimated the weight by a mean of 6.5 kg (21.8%, *P* < 0.001) with the alsg and Luscombe formulas performing best. In 11-12 year old children the alsg formula estimated well. *Conclusion*. In one- to ten-year-old children, the Luscombe formula provided a better weight estimate than alsg and ERC formulas in a multiethnic population.

## 1. Introduction

In a life threatening emergency, rapid establishment of a patient's weight is required to enable calculation of drug doses, amount of fluid to be administered to correct hypovolemic shock, and amount of electricity to be applied in ventricular fibrillation and other arrhythmias. In most cases a present weight is not known and the patient cannot be weighed because of ongoing procedures required for resuscitation, trauma requiring immobilization, and risk of exacerbation of pain. Formulas are therefore used to calculate weight rapidly from age. It is essential to establish which formula is most appropriate in any population at a given time before its application in an emergency. Childhood obesity has been increasing over the years [1, 2] and requires regular appraisal of the appropriateness of formulas for calculation of weight from age. The child heart and health study in England (CHASE study) has found differences in adiposity levels in different ethnic groups with South Asian children having higher obesity levels compared with Caucasian children [3]. In an Asian population in Karnataka, India, the formula used by the European Resuscitation Council (ERC formula, former APLS formula) overestimated the weight by at least 2-3 kg in children one to twelve years of age [4]. In a predominantly black African population in South Africa the ERC formula appeared to provide better accuracy in weight estimation than the Luscombe formula which over estimated weight by 12.4% [5]. The differences in adequacy of weight estimates from formulas in different ethnic groups underscores the need for audits in all communities before the formulas are applied in paediatric emergencies. In 2011 the advanced life support group of the United Kingdom published new formulas for calculation of weight from age [6]. 

The formulas have been developed in response to the previously used APLS formula (the current ERC formula: (age + 4) × 2) underestimating weight significantly by between 18 and 19% in previous studies [7, 8].

Objectives of this prospective audit were as follows.

1. To check the adequacy of the new APLS formula for weight calculation in all age groups.
2. To compare the adequacy of the new APLS formula with other commonly used formulas for weight calculation.
3. To check whether performance of formulas for weight calculation is affected by ethnic group and gender.

## 2. Methods

A prospective audit was conducted on all children aged 1 month to 12 years attending the paediatric assessment unit of the Luton & Dunstable Hospital NHS Foundation Trust between October and December 2011. Data were collected on weight, age, gender, and ethnic group including black African, Asian, and Caucasian groups. The project did not require ethical approval or consent because it fulfilled the criteria for clinical audit set by the National Research Ethics Service of the National Patient Safety Agency for clinical audit including design and conduct to produce information to inform delivery of best care and evaluation of service delivery against a standard [9]. The formulas used were the APLS formula from 2011 (APLS 2011) [6]: 1–11 months: (0.5x age in months) + 4, 1–5 years: (2x age in years) + 8, and 6–12 years: (3x age in years) + 7; the formula previously used by the alsg and currently used by the European Resuscitation Council (ERC) for weight calculation in 1–10 old children [10]: (age + 4) × 2; the Argall formula for 1–10 year olds [7]: (3x age in years) + 6; the Luscombe formula for 1–10 year olds [8]: (3x age in years) + 7; Best Guess [11] formulas: 1–11 months: (0.5x age in months) + 4.5, 1–5 years: 2x (age in years + 5), and 6–14 years: 4x age in years.

The mean differences in weight between actual weight and weight calculated by a formula were represented as percentage with 95% confidence intervals. Data analysis was by *t*-test and calculation of the Pearson correlation coefficient to represent correlation between age and percentage weight difference for the ERC formula. The statistical program used was SPSS release 18.0. A *P* value of <0.05 was taken as indicator of statistical significance.

## 3. Results

We included prospectively 599 children aged 1 month to 12 years of age. There were 157 Asian, 268 Caucasian, and 174 children from other ethnic and mixed origin. 

In infants (*n* = 184) the APLS 2011 and Best Guess formulas didn't calculate a significantly different weight from the actual weight with a mean weight difference of 0.27 kg (95% CI −0.12 to 0.65, *P* = 0.176) for the APLS 2011 and overestimation of −0.23 kg (95% −0.62 to 0.14, *P *= 0.2) for the Best Guess formula. There was no difference between actual and calculated weight for infants (*n* = 27) who were born prematurely (corrected age was used for calculation).

In the age group 1 to 5 years old there was a significant difference between weight calculated by ERC and APLS 2011 formulas (identical for this age group), Argall and Best Guess formulas, and actual weight with children's weight being underestimated by a mean of 1.3 kg (95% CI 0.75 to 1.9, (*P* < 0.001)) for ERC/APLS 2011 formula and for the Argall formula by 0.93 kg (95% CI 0.25 to 1.6, *P* = 0.007). For the Best Guess formula the weight was overestimated by 0.7 kg (95% CI 0.09 to 1.3, *P *= 0.02). There was no significant difference between actual weight and weight calculated by the Luscombe formula (*P* = 0.84) (see Table 1).

**Figure 1:**
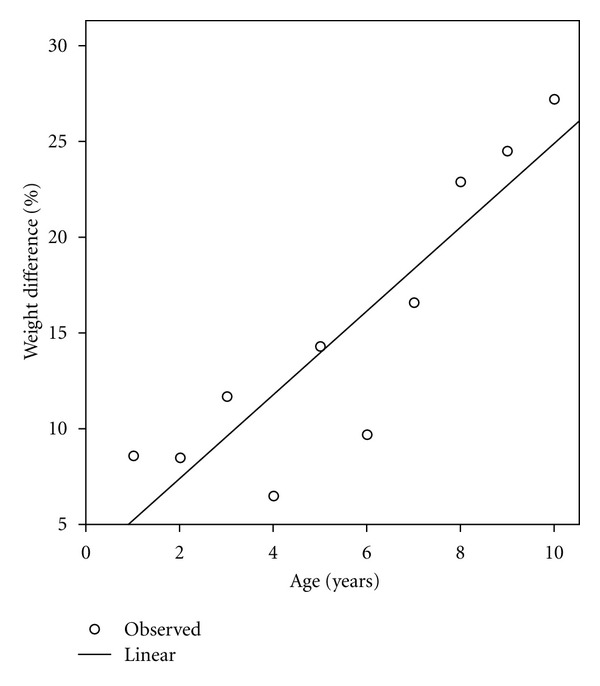
Percentage weight difference between weight calculated by ERC formula (identical to the APLS 2011 formula in the age group 1 to 5 years of age) and actual weight plotted against age (circles represent the percentage points for all children and the line a linear regression line).

To check whether a particular age group, gender, or ethnic group was particularly affected by the underestimation by the APLS 2011 and ERC formulas in this age group (1–5 y), year by year analysis and analysis for ethnic group and gender were performed. For black African children (*n* = 24) there was no significant difference, for Asian children (*n* = 98) these formulas underestimated weight by a mean of 1.0 kg (95% CI 0.13 to 2.0, *P* = 0.02), for Caucasian children (*n* = 114) weight was underestimated by 1.3 kg (95% CI 0.4 to 2.2, *P *= 0.004). In both male and female children weight was significantly underestimated by a similar amount of a mean of 1.3 and 1.4 kg, respectively, (*P* < 0.01). There as a significant under estimation (*P* < 0.01) of weight in each year group from one to five years of age ranging from a mean difference of 1.0 kg (9.6%) in one year olds to a mean difference of 3.1 kg (14.7%) in 5 year olds (see Figure 1).

For the 6–10 age group the ERC formula underestimated weight significantly by a mean of 6.5 kg (95% CI 4.5 to 8.6, *P* < 0.001) while the other formulas yielded no significant weight difference. Calculated weight was significantly underestimated by the ERC formula for the audited childhood population as a whole (see also Table 1 and Figure 1).

For the age group 1 to 10 years of age the relationship between increasing age and underestimation of weight by ERC formula (and for 1–5 year group APLS 2011) was explored. A significant correlation of age with underestimation of weight with *r* = 0.75 (*P *= 0.01) for Asian and *r* = 0.89 (*P* = 0.001) for Caucasian children was found.

For the 11-12 year age group there was no significant difference for calculated versus actual weight using the new APLS 2011 formula (*P *= 0.08) or the Best Guess formula (*P *= 0.64).

## 4. Discussion

The study presented is the first to validate the new APLS formula published 2011. It is the first study investigating formulas used for calculation of weight in a multiethnic population of the United Kingdom.

The new APLS formula published 2011 underestimated weight with the underestimate increasing progressively with age in the 1 to 5 year age group. In this age group it is identical with the ERC formula, that is, the previous APLS formula (see Figure 1). 

We confirmed the result of a previous study [8] that the ERC formula, that is, the formula currently recommended by the European Resuscitation Council progressively underestimates weight with increasing age to an extent which may lead to significant under dosing (by up to 25%) of drugs. Underestimation of weight by the APLS 2011 and ERC formulas is likely related to a progressive increase in excess body fat with age. Some of the drugs used in emergencies like phenytoin used in status epilepticus and propofol used for rapid anesthesia have a significant distribution in fat tissue requiring increase in dose with increasing weight in obese people to achieve effective plasma levels. The loading dose of opioids used for analgesia and anesthesia should also be based on total body weight in obese patients because of a significant distribution in fat tissue [12].

As shown in previous studies [7, 8, 13] in the United Kingdom and Australia, the Luscombe formula provided the most accurate weight estimate across the age group one to ten years in this UK based project in a multiethnic population and was superior to the new APLS 2011 formula. 

## 5. Conclusions

In one- to ten-year-old children, the Luscombe formula provided a better weight estimate than advanced life support group and European Resuscitation Council (ERC) formulas in a multiethnic population in the United Kingdom. The ERC formula should be replaced by the Luscombe formula for the age group between one to ten years of age. Outside this age range the APLS 2011 or Best Guess formulas should be used to provide optimal accuracy of weight estimation.

## Figures and Tables

**Table 1:** Mean differences in percentage (95% confidence interval) of actual weight for formulas used for weight calculation (NA: not applicable).

Age group			Formula		
	APLS 2011^1^	ERC^2^	Luscombe^3^	Argall^4^	Best Guess^5^
1–11 months (*n* = 184)	3.9 (−1.8 to 9.7)	NA	NA	NA	−3.4 (−9.2 to 2.0)
1–5 years (*n* = 275)	9.4 (5.3 to 13.4)	9.4 (5.3 to 13.4)	0.5 (−5.2 to 4.2)	6.6 (1.8 to 11.3)	−4.7 (−8.8 to −0.68)
6–10 years (*n* = 102)	0.42 (−7.1 to 7.2)	21.8 (15.5 to 29.6)	0.12 (−7.1 to 7.2)	3.5 (−3.7 to 10.7)	−1.8 (−8.6 to 5.9)
11-12 years (*n* = 38)	8.0 (−1.2 to 17.1)	NA	NA	NA	−2.1 (−11.4 to 7.2)

^1^1–11 months: (0.5x age in months) + 4; 1–5 years: (2x age in years) + 8; 6–12 years: (3x age in years) + 7 ^2^(age in years +4) x2; ^3^(3x age in years) + 7; ^4^(3x age in years) + 6; ^5^1–11 months: (0.5x age in months) + 4.5; 1–5 years: 2x (age in years + 5); 6–14 years: 4x age in years.
